# The Internationalisation of Graduate Study

**Published:** 1909-09-18

**Authors:** 


### The Internationalisation of Graduate Study.

If the recently held International Congress of Medicine has accomplished nothing else besides the formation of an international committee to look after the interests of graduate students at the various centres, it will have achieved a measure of success which none of its predecessors can be held to have exceeded. For, in a sense, the resolutions which were direct results of Professor Kutner's eloquent appeal for the internationalisation of graduate study mark an epoch in the advance of the graduate study movement. It is, therefore, gratifying to find that England is adequately and worthily represented on the committee that has been formed, and it is sincerely to be hoped that in the near future her representatives will prove, by their energetic support of the various steps that will have to be taken to make the committee an effective practical working organisation and not a mere theoretical nucleus, that they are fully alive to the importance of the graduate study movement. The appointment of this International Committee is the realisation of a splendid hope that Professor Kutner, who has so whole-heartedly worked for the interests of the post-graduate student, expressed several years ago on the occasion of the opening of that magnificent institution on the Luizenplatz, which is the palace of post-graduate study on the Continent. No one who has followed the movement and its various phases—the keen, interested activity of America; the State-supported enthusiasm of Russia; the quiet, serious endeavours of Italy; and the sporadic, isolated attempts of Austria and France—can have failed to be struck with the fact that hitherto England has done comparatively little in the matter of graduate study. At the same time, while that reflection is disheartening, it is an encouraging sign to notice that such graduate-study institutions as we possess are, so far as they go, among the best of their kind. With all their limitations they serve a useful purpose, and, we believe, are growing in popularity. That is as it should be. The graduate-study movement, as we have more than once pointed out, is a matter of national importance, and the sooner the public realises that there can be no finality in the education of members of so catholic a profession as that of medicine, the better it will be for the graduate movement in England. It is perhaps not yet superfluous emphatically to protest against the short-sightedness that sees in post-graduate work a sign of ignorance or of inferiority in the student. That is an opinion still, unfortunately, held by many, and it needs continual protest and opposition to be eradicated. There can be little question that the formation of this International Committee will do much to dispel it by showing, not merely the members of the profession—who are fully alive to it—but the public at large, that it is imperative for the doctor to be a student all his life long. Nor can it be doubted that the committee will do much good work in drawing the attention of the profession to the need for better organisation and greater centralisation of the movement in England. The mere fact that such a committee exists and that it is representative and active will greatly further the cause of the graduate student in this country. There is much work for the committee to do, and it will be interesting to notice how that work will be dealt with and approached. Germany is fortunate in possessing enthusiasts, such as Professor Kutner, who have thrown themselves heart and soul into the movement and who have accomplished success after years of hard endeavour. In America the movement is on a sure foundation, its institutions endowed and possessed of a reputation which is equal to that of our best-known general hospitals. Elsewhere, too, in Russia and Italy, the profession as a whole has shown that it is profoundly interested in what is being done. It remains for England to show what can be done here. The newly-appointed committee will have to make the graduate facilities that are offered by each country available for all countries; and it is imperative, therefore, that it should find the facilities that are offered by institutions in this country such as are worthy of being recommended to the attention of graduate students the world over.

